# Legal liabilities in research: early lessons from North America

**DOI:** 10.1186/1472-6939-6-4

**Published:** 2005-06-13

**Authors:** Randi Zlotnik Shaul, Shelley Birenbaum, Megan Evans

**Affiliations:** 1 Bioethics Department, The Hospital for Sick Children, The University of Toronto Joint Centre for Bioethics, Toronto, Canada; 2 Shelley R. Birenbaum Barrister & Solicitor, Toronto, Canada; 3 Cassels Brock & Blackwell LLP, Toronto, Canada

## Abstract

The legal risks associated with health research involving human subjects have been highlighted recently by a number of lawsuits launched against those involved in conducting and evaluating the research. Some of these cases have been fully addressed by the legal system, resulting in judgments that provide some guidance. The vast majority of cases have either settled before going to trial, or have not yet been addressed by the courts, leaving us to wonder what might have been and what guidance future cases may bring. What is striking about the lawsuits that have been commenced is the broad range of individuals/institutions that are named as defendants and the broad range of allegations that are made. The research community should take this early experience as a warning and should reflect carefully on practices where research involving human subjects is concerned.

## 

The legal risks associated with health research involving human subjects have been highlighted recently by a number of lawsuits launched against those involved in conducting and evaluating the research [1]. Some of these cases have been fully addressed by the legal system, resulting in judgments that provide some guidance. The vast majority of cases have either settled before going to trial, or have not yet been addressed by the courts, leaving us to wonder what might have been and what guidance future cases may bring. What is striking about the lawsuits that have been commenced is the broad range of individuals/institutions that are named as defendants and the broad range of allegations that are made.

## Plaintiffs cast a wide net: the range of defendants named

A review of recent Canadian and American cases demonstrates that in commencing lawsuits over alleged research misconduct, plaintiffs cast a wide net, naming as defendants anyone who had anything to do with the research in question. Named defendants have included the researchers, the research ethics committee/board/institutional review board (REC) that approved the research and its individual members, as well as bioethicists who consulted on the research project.

For example, in *Gelsinger *v. *Trustees of the University of Pennsylvania *[2], an eighteen year-old who had volunteered to participate in a corrective gene study died during the course of the study. In that case, the trustees of the university and two hospitals affiliated with the research, the investigators, the company that sponsored the research, the former medical school dean and a bioethicist, were all originally named as defendants on the bases (among others) of wrongful death, assault and battery linked to a lack of informed consent, and common law fraud/misrepresentation linked to the informed consent process. The case settled for an undisclosed amount [3].

In *Robertson *v. *McGee *[4], the REC had approved a protocol for a Phase I study of a cancer vaccine. Many of the patients who enrolled in the study had advanced disease, were unresponsive to standard therapies and had very poor prognoses. According to news reports, 94 subjects received the vaccine and 26 died during the study, although the deaths were not attributed to the vaccine itself [5]. On January 29, 2001, a number of subjects and subject representatives filed a lawsuit seeking actual and punitive damages. The *Robertson *plaintiffs sued the hospital, the principal investigator, the pharmaceutical sponsor, a top university official, the individual members of the REC, and the university bioethicist who consulted with the REC. The issues in this case were never decided by the court because the court held that it did not have jurisdiction over the allegations made in the complaint and dismissed the case.

In the case of *Weiss *v. *Solomon *[6], a research subject suffered a cardiac arrest and died after undergoing a fluorescein angiogram as part of a research study. The deceased's family sued the principal investigator, the hospital and a physician who referred one of his patients to the study. The Court hearing the case ultimately found liability against the primary investigator and hospital only. Interestingly, the liability against the hospital was based in part upon the fact that the hospital's REC had approved the research protocol and the consent form which was determined by the Court to be deficient.

All of these cases demonstrate that plaintiffs cast a wide net when deciding whom to name as defendants in cases where research goes wrong. It is possible for almost anyone involved in a research project, no matter how remotely connected, to be named in legal proceedings if something goes wrong. Unfortunately, there are not enough decided cases to predict with any degree of certainty how far the courts in Canada and the United States (U.S.) will be prepared to go in attributing fault in research negligence cases to those that have less than a direct connection to the injuries that have been sustained.

## Broad range of allegations made

A review of the lawsuits that have been commenced recently demonstrates that there are a broad range of allegations that can potentially be made against those involved in research. The allegations are such that it is open to a plaintiff to sue not only the primary investigator and others directly involved in the research but also those with a much less tangible relationship to the research subject. What follows is a brief overview of some of the more common allegations that have been made and what, if anything, the courts have said about these allegations.

### Deficiencies in the consent process

It is perhaps obvious for most health care professionals that liability will arise where research is conducted without informed consent from the subjects (or their substitute decision makers) being obtained. What may be less known is that it appears from existing case law as though the standard for obtaining informed consent in the research context is higher than in the therapeutic context. The other interesting development has been that it appears that courts may be willing to hold a hospital responsible where the informed consent form approved by the REC of the Hospital is determined to be less than adequate.

In the *Weiss *case for example, the Quebec Superior Court found that the duty to inform in matters relating to purely scientific experimentation is the most exacting possible and includes the disclosure of all known risks including those which are rare or remote, especially if they may entail grave consequences [7]. This would suggest a standard that is higher than the standard for consent to treatment which requires that only material risks be disclosed.

In addition, the court in Weiss found that the hospital was liable and attributed some of that liability to the fact that the hospital's REC failed to ensure that the consent form used for the research was appropriate.

### Failure to follow laws, regulations, policies, procedures and guidelines

Some of the recent lawsuits have alleged that the defendants failed to follow applicable laws, regulations, policies, procedures and/or guidelines. If these allegations can be proven, it is likely that liability will follow, as a failure to comply with applicable laws, regulations, policies, procedures and/or guidelines will likely be interpreted by the courts as a clear sign that the defendants failed to meet the standard of care.

### Government initiated legal proceedings

In addition to being sued by research subjects who allege being injured by their participation in a study, there is also a possibility that the researchers and institutions involved in health research involving human subjects will face legal battles with governmental bodies/agencies with jurisdiction over research. In the U.S., such bodies have imposed drastic sanctions where there is evidence of research misconduct.

The defendants in the *Gelsinger *case not only settled with the plaintiffs, they also settled with the U.S. government in a separate civil action commenced by the government on the basis of breach of the *False Claims Act *[8]. The settlement resulted in a total of over one million dollars in payments to the government by the institutions involved in the research as well as restrictive controls being placed on three investigators involved in the research with respect to their future clinical research activities [9].

## Conflict of Interest

In the case of *Gelsinger*, the complaint alleged, among other things, that the university was to receive an ownership stake in Genovo (the sponsor) in lieu of funding of the gene transfer research program and that the University and various physicians associated with the research program had substantial financial and equity interests with respect to the vectors employed in the research [10]. The extent of these financial interests were not disclosed to Jesse Gelsinger before he made his decision to participate in the research as a subject [11]. If this case had not settled and instead proceeded to a trial, one of the key issues would have been the circumstances under which an undisclosed conflict of interest will result in liability for the individual(s) and/or entities who are in the conflict position and whether other parties who know of the conflict or who ought reasonably to know about the conflict and are in a position of authority have a legal obligation to intervene to prevent the research from proceeding on the basis of the conflict. The existence of an undisclosed conflict of interest may also put a plaintiff in the position of being able to argue that the consent given was not truly informed.

## Lawsuits based on principles of international human rights

In the case of *Abdullahi *v. *Pfizer, Inc*. [12], Pfizer was the defendant in a lawsuit that alleged that it improperly administered an experimental antibiotic to children in Nigeria during an outbreak of bacterial meningitis, measles and cholera in Kano, Nigeria. The guardians of certain of those children instituted an action in the Southern District of New York, alleging violations of the Nuremberg Code, the Declaration of Helsinki, Article 7 of the International Covenant on Civil and Political Rights, U.S. Food and Drug Administration's regulations and other norms of international law. They also asserted that the court had jurisdiction over the matter under the *Alien Torts Claim Act*. This case was sent back to the District Court to determine whether the U.S. or Nigeria is the appropriate forum to hear the case [13].

In the *Robertson *case, several of the causes of action were derived from international human rights law rather than standard medical malpractice or tort law, including allegations of breaching the right to be treated with dignity, citing the Nuremberg Code and Declaration of Helsinki concerning biomedical research [14].

## Conclusion

Admittedly, the number of judgments rendered in research misconduct lawsuits in North America to date is small. That being said, experience to date demonstrates that:

(a) plaintiffs will likely cast a wide net, naming anyone or any institution that had even the slightest involvement or connection to the research;

(b) plaintiffs have found a number of different ways to frame their claims;

(c) the Government may be a potential plaintiff in these lawsuits; and

(d) the standards applicable in the research context may, in fact, be higher than those applicable in the therapeutic treatment context.

The research community should take this early experience as a warning and should reflect carefully on practices where research involving human subjects is concerned.

## Competing interests

The author(s) declare that they have no competing interests.

## Pre-publication history

The pre-publication history for this paper can be accessed here:


